# Duodenal and faecal microbiota of celiac children: molecular, phenotype and metabolome characterization

**DOI:** 10.1186/1471-2180-11-219

**Published:** 2011-10-04

**Authors:** Raffaella Di Cagno, Maria De Angelis, Ilaria De Pasquale, Maurice Ndagijimana, Pamela Vernocchi, Patrizia Ricciuti, Francesca Gagliardi, Luca Laghi, Carmine Crecchio, Maria Elisabetta Guerzoni, Marco Gobbetti, Ruggiero Francavilla

**Affiliations:** 1 Department of Biologia e Chimica Agro-Forestale ed Ambientale, University of Bari Aldo Moro, via Amendola 165/A, Bari, 70126 Italy; 2 Department of Food Science, Alma Mater Studiorum, University of Bologna, via Fanin 46, Bologna, 40127 Italy; 3 Department of Pediatrics, University of BariAldo Moro, P.zza Giulio Cesare 11, Bari, 70126 Italy

## Abstract

**Background:**

Epidemiology of celiac disease (CD) is increasing. CD mainly presents in early childhood with small intestinal villous atrophy and signs of malabsorption. Compared to healthy individuals, CD patients seemed to be characterized by higher numbers of Gram-negative bacteria and lower numbers Gram-positive bacteria.

**Results:**

This study aimed at investigating the microbiota and metabolome of 19 celiac disease children under gluten-free diet (treated celiac disease, T-CD) and 15 non-celiac children (HC). PCR-denaturing gradient gel electrophoresis (DGGE) analyses by universal and group-specific primers were carried out in duodenal biopsies and faecal samples. Based on the number of PCR-DGGE bands, the diversity of *Eubacteria *was the higher in duodenal biopsies of T-CD than HC children. *Bifidobacteria *were only found in faecal samples. With a few exceptions, PCR-DGGE profiles of faecal samples for *Lactobacillus *and *Bifidobacteria *differed between T-CD and HC. As shown by culture-dependent methods, the levels of *Lactobacillus*, *Enterococcus *and *Bifidobacteria *were confirmed to be significantly higher (*P *= 0.028; *P *= 0.019; and *P *= 0.023, respectively) in fecal samples of HC than in T-CD children. On the contrary, cell counts (CFU/ml) of presumptive *Bacteroides*, *Staphylococcus*, *Salmonella*, *Shighella *and *Klebsiella *were significantly higher (*P *= 0.014) in T-CD compared to HC children. *Enterococcus faecium *and *Lactobacillus plantarum *were the species most diffusely identified. This latter species was also found in all duodenal biopsies of T-CD and HC children. Other bacterial species were identified only in T-CD or HC faecal samples. As shown by Randomly Amplified Polymorphic DNA-PCR analysis, the percentage of strains identified as lactobacilli significantly (*P *= 0.011) differed between T-CD (ca. 26.5%) and HC (ca. 34.6%) groups. The metabolome of T-CD and HC children was studied using faecal and urine samples which were analyzed by gas-chromatography mass spectrometry-solid-phase microextraction and ^1^H-Nuclear Magnetic Resonance. As shown by Canonical Discriminant Analysis of Principal Coordinates, the levels of volatile organic compounds and free amino acids in faecal and/or urine samples were markedly affected by CD.

**Conclusion:**

As shown by the parallel microbiology and metabolome approach, the gluten-free diet lasting at least two years did not completely restore the microbiota and, consequently, the metabolome of CD children. Some molecules (e.g., ethyl-acetate and octyl-acetate, some short chain fatty acids and free amino acids, and glutamine) seems to be metabolic signatures of CD.

## Background

Celiac disease (CD) is the chronic gastrointestinal (GI) tract disorder where ingestion of gluten from wheat, rye and barley, and their cross related varieties, leads to damage of the small intestinal mucosa by an autoimmune mechanism in genetically susceptible individuals [1]. Epidemiology of CD is increasing, the prevalence is estimated to be ca. 1% in the European and North American populations [1,2]. CD mainly presents in early childhood with small intestinal villous atrophy and signs of malabsorption [3]. Nowadays, the gluten-free diet (GFD) is the only effective and safe treatment for CD. Nevertheless, compliance with this dietary therapy is very complex and patients may suffer of health risks and nutritional deficiencies [4,5]. Recently, some reports also suggested that the GI microbiota is somewhat affected during CD pathogenesis and GFD [6-10].

The human GI tract is a complex ecosystem integrated by up to 10^14 ^total bacteria. The genomes of all intestinal microbes form the "microbiome", representing more than 100 times the human genome. This latter, in association with the microbiome, is considered as the "metagenome" [11]. As the consequence, the microbiome provides the human host with additional metabolic functions, described as the "metabolome".

Some of the main activities provided by the gut microbiota in human health are: (i) to provide a barrier for colonization of pathogens; (ii) to exert important metabolic functions such as fermentation of non-digestible fibers, salvage of energy as short chain fatty acids (SCFA) and synthesis of vitamin K; and (iii) to stimulate the development of the immune system [12]. Besides, specific strains of the GI microbiota and/or supplied probiotics decrease intestinal inflammations and normalize dysfunctions of the GI mucosa [13,14]. Indeed, GI microbiota is also involved in the pathogenesis of chronic inflammatory bowel diseases (IBD) and other immune-related disorders [15]. Overall, IBD patients have altered densities of mucosa-associated bacteria (of duodenal bacterial population) in comparison to healthy subjects. In particular, cell numbers of protective *Bifidobacterium *and *Lactobacillus *decreased, while harmful *Bacteroides *and *Escherichia coli *increased [15]. Recently, microbial infections and, especially, imbalances of the composition of the GI microbiota were associated with the presentation of CD also [7-10,16]. Compared to healthy individuals, CD patients seemed to be characterized by higher numbers of Gram-negative bacteria and lower numbers Gram-positive bacteria [10,16]. Overall, Gram-negative bacteria could activate pro-inflammatory pathways, while Gram-positive bacteria such as lactic acid bacteria and bifidobacteria could inhibit toxic effects induced by other GI species [17] or gluten antigens [18,19]. Duodenal and faecal bacterial populations, especially *Bifidobacteria*, significantly varied within individuals, being influenced either by diet or CD [20,21]. The composition of *Lactobacillus *sp. and *Bifidobacterium *species differed between CD patients and healthy children [9]. Recent studies indicated that CD patients at diagnosis or under GFD had unbalanced serum, faecal and urine metabolites [10,22]. It was hypothesized that qualitative and quantitative differences of the microbiota influenced the level of volatile organic compounds (VOC) of CD patients [10]. More in depth characterization of the GI microbiota and related metabolites is strongly needed for CD patients and the role of bacteria during CD development and treatment has to be elucidated [8,9,23].

This study aimed at comparing the differences of the microbiota and metabolome between CD children under GFD (treated celiac disease, T-CD) and non-celiac children (healthy control, HC). The intestinal and faecal microbiota was characterized by culture-independent and -dependent methods whereas metabolomic studies were carried out using gas-chromatography mass spectrometry/solid-phase microextraction (GC-MS/SPME) and ^1^H nuclear magnetic resonance (NMR) spectroscopy.

## Results

### Molecular analysis of the bacterial community of duodenal biopsies and faecal samples

**Figure 1:**
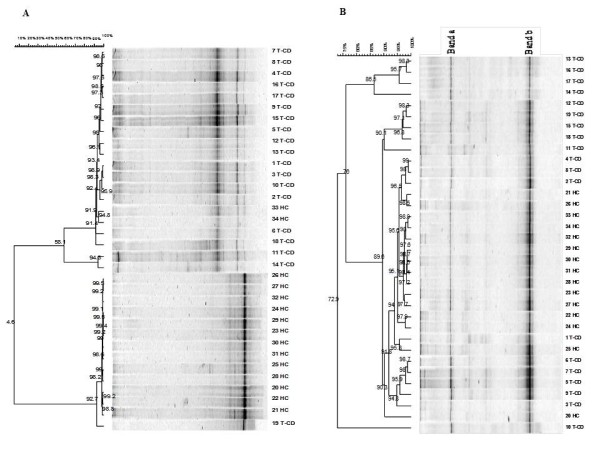
**Clustering of denaturing gradient gel electrophoresis (DGGE) profiles of biopsies from thirty-four children (1-34)**. Universal V6-V8 (A) and Lac1/Lac2 *Lactobacillus *group (B) primers were used. Clustering was carried out using the unweighted pair-group method with the arithmetic average (UPGMA) based on the Pearson correlation coefficient. T-CD, treated celiac disease children; and HC, non-celiac children; band a, *L. plantarum*; band b, human DNA. See materials and methods for correspondence of numbered duodenal biopsies.

The dominant microbiota and specific subgroups (*Bifidobacteria *and *Lactobacillus*) from stool samples and from duodenal biopsies (mucus and mucosa associated bacteria) were analyzed by PCR (Polymerase chain reaction)-DGGE (denaturing gradient gel electrophoresis). Universal primers targeting V6-V8 regions of the 16S rRNA gene were used. Eubacterial profiles from PCR-DGGE analysis of duodenal biopsies of treated celiac disease (T-CD) children showed high richness with two to eight well resolved and strong bands (Figure 1A). Only the electrophoretic profile of 19 T-CD duodenal biopsy contained one band. Profiles of non-celiac children (HC) had only one to three strong bands. Banding patterns were processed using the Bionumerics software. Pearson correlation coefficients ranged from 4.6 to 99.5%. Except for two duodenal biopsies (33 and 34 HC) which showed high similarity to T-CD samples, all HC banding patterns were grouped together with 98.2% similarity coefficient. The major part of the T-CD samples were grouped together at 95% of the similarity. Overall, DGGE profiles of the PCR amplicons obtained with primers Lac1 and Lac2 had two strong, common and well-resolved bands, and a few bands with low intensity (Figure 1B). High similarity was found among samples belonging to T-CD and HC groups. Most of the T-CD and HC duodenal biopsies were grouped together at ca. 90% of similarity and all samples at 72.9%. Sequencing of the DGGE bands revealed the common presence of *L. plantarum *(band a). Although Lac1 and Lac2 primers were commonly used to detect *Lactobacillus *species [9,24,25], human DNA (band b) was also found. Finally, no PCR amplicons were found by using three different sets of primers targeting the *Bifidobacteria *group. This suggested that *Bifidobacteria *were probably absent from duodenal biopsies of both T-CD and HC.

**Figure 2:**
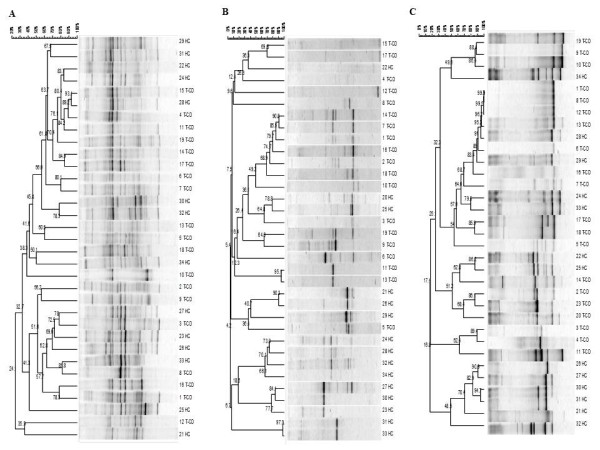
**Clustering of denaturing gradient gel electrophoresis (DGGE) profiles of faecal samples from thirty-four children (1-34)**. Universal V6-V8 (A), Lac1/Lac2 *Lactobacillus *group (B), g- Bifid F/g-BifidRGC *Bifidobacterium *group (C) primers were used. Clustering was carried out using the unweighted pair-group method with the arithmetic average (UPGMA) based on the Pearson correlation coefficient. T-CD, treated celiac disease children; and HC, non-celiac children. See materials and methods for correspondence of numbered faecal samples.

Compared to duodenal biopsies, the PCR-DGGE profiles of faecal samples were more rich. Although fingerprints contained many well-resolved and strong bands, unresolved bands or very weak separate fragments were present in some regions of the gel. The PCR-DGGE profiles from universal primers (Table 1) targeting V6-V8 regions of the 16S rRNA gene were very rich in bands quite different for each of the 34 children (Figure 2A). Only some common bands were present. The uniqueness of the patterns was confirmed by cluster analysis. The values of Pearson similarity were always low. The mean similarity coefficient was 24.1%. No clustering differentiated T-CD and HC samples. Figure 2B shows the PCR-DGGE profiles from primers Lac1 and Lac2 specific for *Lactobacillus *group. Depending on the faecal sample, one to four strong and well-resolved amplicons were detected. Nevertheless, the values of Pearson similarity coefficient were low and all samples grouped together at ca. 4.2%. According to PCR-DGGE profiles of duodenal biopsies, the UPGMA clusterization grouped separately T-CD and HC samples with the only exceptions of sample 5 T-CD coupled to HC, and samples 22, 20 and 25 HC which showed high similarity to T-CD. Anyway significant differences were present within groups of T-CD or HC children.

**Table 1:** Primers used and conditions for denaturing gradient gel electrophoresis (DGGE) analysis

Primer	Primer sequence (5'-3')	Amplicon size (bp)	Annealing temperature (°C)	DGGE gradient (%)	Target group	Reference
V6-V8: F968-GC V6-V8: R1401	GC clamp^a^-AACGCGAAGAACCT CGGTGTGTACAAGACCC	489	55	45-55 (feces) 40-65 (biopsies)	Eubacteria	This study
g- Bifid F g-Bifid R-GC	CTCCTGGAAACGGGTGG GC clamp^a^-GGTGTTCTTCCCGATATCTACA	596	65	45-60	*Bifidobacterium*	This study
Lac1 Lac2GC	AGCAGTAGGGAATCTTCCA GC clamp^a ^- ATTYCACCGCTACACATG	380	61	35-50 (feces) 35-70 (biopsies)	*Lactobacillus *group^b^	[24]
Bif164-f Bif662-GC-r	GGGTGGTAATGCCGGATG GC clamp ^a^- CCACCGTTACACCGGGAA	520	62	45-55	*Bifidobacterium*	[47]
Bif164-GC-f Bif662-r	GC clamp ^a ^- GGGTGGTAATGCCGGATG CCACCGTTACACCGGGAA	520	62	45-55	*Bifidobacterium*	[47]

^a^GC clamp sequence: CGCCCGCCGCGCCCCGCGCCCGGCCCGCCGCCCCCGCCCC.

^b^*Lactobacillus *group comprises the genera *Lactobacillus*, *Leuconostoc*, *Pediococcus *and *Weisella*.

As shown by PCR-DGGE analysis, all faecal samples contained *Bifidobacterium *DNA (Figure 2C). The level of similarity among faecal samples varied from 16.8 to 100%. Identical profiles were found for some T-CD stool samples (numbers 1, 8 and 12). The UPGMA analysis grouped most of T-CD and HC profiles separately, with similarity Pearson coefficients ≥ 48%.

### Enumeration of cultivable bacteria

**Figure 3:**
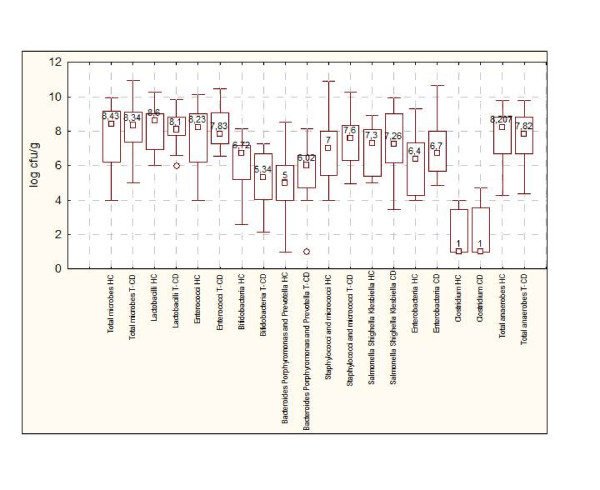
**Cultivable cells (log cfu/g) of the main microbial groups in faecal samples of treated celiac disease (T-CD) children and non-celiac children children (HC)**. The data are the means of three independent experiments (n = 3). The top and bottom of the box represent the 75th and 25th percentile of the data, respectively. The top and bottom of the error bars represent the 5th and 95th percentile of the data, respectively.

Selective media were used to enumerate cultivable cells of the main microbial groups (Figure 3). No statistical difference (*P *= 0.161) was found between T-CD and HC for total microbes. The median values of presumptive lactobacilli and enterococci of T-CD was lower (*P *= 0.035) than those of HC. The number of presumptive *Bifidobacteria *significantly (*P *= 0.023) differed between T-CD (median value of 5.34 ± 0.020 log CFU/g) and HC (median value of 6.72 ± 0.023 log CFU/g). Compared to HC, significantly (*P *= 0.014) higher counts of presumptive *Bacteroides*, *Porphyromonas *and *Prevotella*, presumptive staphylococci/micrococci and *Enterobacteria *were found in faecal samples of T-CD. Presumptive *Salmonella, Shighella *and *Klesbiella*, and *Clostridium *did not significantly (*P *= 0.830) vary between groups. Total anaerobes were the highest (*P *= 0.018) in HC.

### Identification and typing of lactic acid bacteria

Colonies of presumptive lactic acid bacteria were randomly isolated from the highest plate dilutions of MRS or Blood Azide agar and used for further analysis. Gram-positive, catalase-negative, non-motile cocci and rods able to acidify MRS or Blood Azide broth (ca. 438 isolates corresponding to ca. 13 isolates per child) were identified by sequence analysis of at least 700 bp of the 5' region of the 16S rRNA gene (Table 2). Discrimination between *Enterococcus faecalis*/*E. faecium*/*Enterococcus durans*, *L. plantarum*/*Lactobacillus pentosus*/*Lactobacillus paraplantarum *or *Lactobacillus paracasei*/*Lactobacillus casei*/*Lactobacillus rhamnosus *was allowed by partial sequencing of *recA *or *pheS *genes. *Enterococcus *was the genus most largely isolated within the lactic acid bacteria group for both T-CD and HC children (Table 2). *E. faecium *was the species identified in almost all faecal samples (13 of 19 and 10 of 15 for T-CD and HC, respectively). *E. avium *(6/19 and 4/15 for T-CD and HC, respectively), *E. faecalis *(3/19 and 2/15 for T-CD and HC, respectively)*, E. durans *(3/19 and 5/15 for T-CD and HC, respectively) and *Enterococcus *spp. (11/19 and 12/15 for T-CD and HC, respectively) were variously identified. *Streptococcus macedonicus *(1/19), *Streptococcus pasteurianus *(1/19), *Pediococcus acidilactici *(4/19) and *Pediococcus pentosaceus *(2/19) were only isolated in T-CD children. *L. plantarum *(4/19 and 12/15 for T-CD and HC, respectively), *L. casei *(5/19 and 5/15 for T-CD and HC, respectively) and *L. rhamnosus *(2/19 and 2/15 for T-CD and HC, respectively) were the species of lactobacilli which were most largely isolated in both T-CD and HC. On the contrary, *Lactobacillus salivarius *(4/19), *Lactobacillus coryneformis *(2/19), *Lactobacillus delbrueckii *subsp. *bulgaricus *(1/19), *Lactobacillus fermentum *(1/19) and *L. paracasei *(1/19) were only identified in faecal samples of T-CD. *Lactobacillus brevis *(1/15)*, Lactobacillus pentosus *(1/15) and *Lactobacillus mucosae *(1/15) were only identified in faecal samples of HC.

**Table 2:** Species of the *Lactobacillus *and *Enterococcus *genera identified in faecal samples by 16S rRNA and pheS or recA gene sequencing

Sample	Number of isolates	**Number of strains identified**^**a**^	Closest relative and identity (%)	Accession Number
Treated celiac disease (T-CD) children				

1	3	3-IV^b^	*Pediococcus pentosaceus *(99%)	[GenBank:FJ844959.1]
	1, 7	1-VII, 5-XI	*Enterococcus faecium *(99%)	[GenBank:FJ982664.1]
	1	1-XII	*Enterococcus avium *(99%)	[GenBank:HQ169120.1]
	1	1-20I	*Lactobacillus plantarum *(99%)	[GenBank:HQ441200.1]
	1	1-7I	*Lactobacillus delbrueckii *subsp. *bulgaricus *(99%)	[GenBank:CP002341.1]
2	12	6-IV	*Pediococcus pentosaceus *(99%)	[GenBank:FJ844959.1]
3	2, 1, 1	2-XIV, 1-6I, 1-1I	*Enterococcus faecium *(99%)	[GenBank:HQ293070.1]
	6	6-XVI	*Enterococcus faecalis *(99%)	[GenBank:HQ293064.1]
	1	1-9I	*Lactobacillus salivarius *(99%)	[GenBank:GU357500.1]
4	1, 3, 2	1-II, 3-V, 2-VII	*Enterococcus faecium *(99%)	[GenBank:HQ293070.1]
	3, 1, 1	3-II, 1-IV, 1-V	*Enterococcus avium *(99%)	[GenBank:HQ169120.1]
	1	1-24I	*Lactobacillus casei *(99%)	[GenBank:HQ379174.1]
	1	1-11I	*Lactobacillus plantarum *(99%)	[GenBank:EF439680.1]
5	5	5-VII	*Enterococcus faecium *(99%)	[GenBank:FJ982664.1]
	1, 3	1-6I, 2-XIX	*Enterococcus sp*. (99%)	[GenBank:AB470317.1]
	1	1-11I	*Lactobacillus rhamnosus *(99%)	[GenBank:HM218396.1]
	1, 1	1-1I, 1-8I	*Lactobacillus fermentum *(99%)	[GenBank:HQ379178.1]
6	5	1(5I-11I-7I-12I-2I)	*Enterococcus avium *(99%)	[GenBank:HQ169120.1]
	4	3-XXII	*Enterococcus sp*. (99%)	[GenBank:AB470317.1]
	1, 1	1-1I, 1-3I	*Lactobacillus plantarum *(99%)	[GenBank:EF439680.1]
7	1	1-12I	*Enterococcus avium *(99%)	[GenBank:HQ169120.1]
	11	4-XX	*Streptococcus macedonicus *(99%)	[GenBank:EU163501.1]
8	1	1-VII	*Enterococcus faecium *(99%)	[GenBank:HQ293070.1]
	1	1-14I	*Enterococcus *sp. (99%)	[GenBank:AB470317.1]
	4, 3, 1, 1, 1, 1	4-III, 3-IV, 1-6I, 1-12I, 1-14I, 1-15I	*Lactobacillus salivarius *(99%)	[GenBank:FJ378897.1]
9	2, 3	1-III,3-IV	*Enterococcus faecalis *(99%)	[GenBank:HQ293064.1]
	1, 1, 1, 3, 1	10I, 1-V, 1-VI, 3-VII, 1-2I	*Enterococcus faecium *(99%)	[GenBank:HQ293070.1]

Treated celiac disease (T-CD) children				

	1	1-14I^b^	*Lactobacillus casei *(99%)	[GenBank:HQ318715.2]
10	1	1-III	*Enterococcus faecalis *(99%)	[GenBank:HQ293064.1]
	1	1-VII	*Enterococcus durans *(99%)	[GenBank:HM218637.1]
	1	1-VII	*Enterococcus faecium *(99%)	[GenBank:HQ293070.1]
	2	2-VIII	*Enterococcus *sp. (99%)	[GenBank:AB470317.1]
	2, 1	2-III, 1-3I	*Lactobacillus salivarius *(99%)	[GenBank:FJ378897.1]
	2	V	*Lactobacillus coryniformis *(99%)	[GenBank:HQ293050.1]
11	1, 1	1-4I, 1-12I	*Enterococcus *sp. (99%)	[GenBank:AB470317.1]
	1	1-8I	*Pediococcus acidilactici *(99%)	[GenBank:GU904688.1]
	1	1-11I	*Enterococcus durans *(99%)	[GenBank:HM218637.1]
	2, 1, 3, 1, 1	2-I, 1-1I, 1(6I, 5I,7I), 1-3I, 1-2I	*Enterococcus faecium *(99%)	[GenBank:U385351.1]
12	10	5-IV	*Pediococcus acidilactici *(99%)	[GenBank:GU904688.1]
	1	1-6I	*Enterococcus *sp. (99%)	[GenBank:AB470317.1]
13	1	3I	*Enterococcus *sp. (99%)	[GenBank:AB470317.1]
	1, 7	1-VII, 3-XVIII	*Enterococcus faecium *(99%)	[GenBank:HQ293070.1]
14	8, 2	4-III, 2-IX	*Enterococcus avium *(99%)	[GenBank:HQ169120.1]
	1	1-IV	*Pediococcus acidilactici *(99%)	[GenBank:GU904688.1]
	2, 1, 1, 2	2-I, 1-22I, 1-III, 2-VI	*Lactobacillus plantarum *(99-100%)	[GenBank:HQ441200.1]
15	8, 1	8-IV, 1-2I	*Pediococcus acidilactici *(99%)	[GenBank:GU904688.1]
	1	1-8I	*Enterococcus *sp. (99%)	[GenBank:AB470317.1]
	1	1-XVIII	*Enterococcus faecium *(99%)	[GenBank:HQ293070.1]
	1	1-III	*Lactobacillus casei *(99%)	[GenBank:HQ379174.1]
16	2	2-X	*Enterococcus faecium *(99%)	[GenBank:AB596997.1]
	2, 8	2-XV, 7-XXI	*Streptococcus pasteurianus *(99%)	[GenBank:AB457024.1]
	3	1(13I-14I-5I)	*Enterococcus *sp. (99%)	[GenBank:AB470317.1]
17	1	1-VI	*Enterococcus faecium *(99%)	[GenBank:AB596997.1]
	8	7-XII	*Enterococcus avium *(99%)	[GenBank:HQ169120.1]
	3, 1	2-XIII, 1-13I	*Enterococcus *sp. (99%)	[GenBank:AB470317.1]
18	6, 6	3-VI, 2-XVII	*Enterococcus faecium *(99%)	[GenBank:AB596997.1]
	1	1-13I	*Enterococcus *sp. (99%)	[GenBank:AB470317.1]
	3	3-II	*Lactobacillus rhamnosus *(99%)	[GenBank:HM218396.1]

Treated celiac disease (T-CD) children				

	1	1-14I^b^	*Lactobacillus casei *(99%)	[GenBank:HQ318715.2]
19	1	1-VII	*Enterococcus durans *(99%)	[GenBank:HM218637.1]
	6	5-III	*Lactobacillus salivarius *(99%)	[GenBank:FJ378897.1]
	2	2-III	*Lactobacillus paracasei *(99%)	[GenBank:HQ423165.1]
	1, 4, 1	24I, 3-III, 23I	*Lactobacillus casei *(99%)	[GenBank:HQ379174.1]
	3	3-V	*Lactobacillus coryniformis *99%)	[GenBank:HQ293050.1]

Heathy children (HC)				

20	3	1-III	*Enterococcus *sp. (99%)	[GenBank:AB470317.1]
	1, 6	1-2I, 3-VII	*Enterococcus avium *(99%)	[GenBank:HQ169120.1]
	2	2-XIII	*Enterococcus faecalis *(99%)	[GenBank:HQ228219.1]
	1	1-6I	*Lactobacillus plantarum *(99%)	[GenBank:EF439680.1]
21	3, 5	3-VI, 4-VII	*Enterococcus avium *(99%)	[GenBank:HQ169120.1]
	2	2-XII	*Enterococcus *sp. (99%)	[GenBank:AB470317.1]
	1, 1	1-3I, 1-XI	*Lactobacillus plantarum *(99%)	[GenBank:EF439680.1]
22	1, 1	1-III, 1-10I	*Enterococcus *sp. (99%)	[GenBank:AB470317.1]
	4	3-VI	*Enterococcus faecium*(99%)	[GenBank:DQ305313.1]
	5	5-VI	*Enterococcus avium *(99%)	[GenBank:HQ169120.1]
	1	1-9I	*Enterococcus durans *(99%)	[GenBank:HM218738.1]
	1	1-XI	*Lactobacillus plantarum *(99%)	[GenBank:EF439680.1]
	1	1-11I	*Lactobacillus mucosae *(99%)	[GenBank:AB425938.1]
23	3	3-III	*Enterococcus *sp. (99%)	[GenBank:AB470317.1]
	4	3-IV	*Enterococcus durans *(99%)	[GenBank:HM218637.1]
	2	2-VI	*Enterococcus faecium*(99%)	[GenBank:FJ982664.1]
	3, 2	3-VI, 2-VII	*Enterococcus avium *(99%)	[GenBank:HQ169120.1]
24	1, 1	1-5I, 1-8I	*Enterococcus faecalis *(99%)	[GenBank:HM480367.1]
	1, 1, 1, 1, 1	1-9I, 1-4I, 1-XVI, 1-7I, 1-1I	*Enterococcus faecium *(99%)	[GenBank:HQ293070.1]
	1, 1	1-XVI, 1-3I	*Enterococcus durans *(99%)	[GenBank:HM218637.1]
	1	1-2I	*Lactobacillus plantarum *(99%)	[GenBank:EF439680.1]
25	3, 1, 1, 1	2-III, 1-V, 1-XIV, 1-2I	*Enterococcus *sp. (99%)	[GenBank:DQ305313.1]
	1	1-VIII	*Enterococcus faecium *(99%)	[GenBank:AB596997.1]

Heathy children (HC)				

	1	1-III^b^	*Lactobacillus casei *(99%)	[GenBank:HQ379174.1]
	3, 1	3-III, 1-XI	*Lactobacillus plantarum *(99%)	[GenBank:EF439680.1]
26	4	3-IX	*Enterococcus *sp. (99%)	[GenBank:DQ305313.1]
	2, 1	2-XI, 1-11I	*Enterococcus faecium *(99%)	[GenBank:FJ982664.1]
	1	1-7I	*Lactobacillus plantarum *(99%)	[GenBank:HQ441200.1]
	1, 2, 1, 1, 1	1-13I, 2-VI, 1-8I, 1-2I, 1-7I	*Lactobacillus casei *(99%)	[GenBank:HQ379174.1]
27	2, 1, 1	1(3I-13I), 1-1I, 1-6I	*Enterococcus *sp. (99%)	[GenBank:DQ305313.1]
	1, 1, 1, 2	1-5I, 1-2I, 1-7I, 2-XVI	*Enterococcus faecium *(99%)	[GenBank:AB596997.1]
	3	2-XV	*Enterococcus durans *(99%)	[GenBank:HM209741.1]
	1	1-11I	*Lactobacillus plantarum *(99%)	[GenBank:EF439680.1]
28	4, 1	4-VIII, 1-1I	*Enterococcus faecium *(99%)	[GenBank:AB596997.1]
	1, 1, 2	1(4I-5I), 2-XIV	*Enterococcus *sp. (99%)	[GenBank:AB470317.1]
	3	2-I	*Lactobacillus plantarum *(99%)	[GenBank:HQ441200.1]
	3	3-II	*Lactobacillus rhamnosus *(99%)	[GenBank:HM218396.1]
	1	1-4I	*Lactobacillus brevis *(99%)	[GenBank:HQ293087.1]
29	1, 1, 1	12I, 1(10I-11I), 1-1I	*Enterococcus *sp. (99-100%)	[GenBank:AB470317.1]
	5, 1, 1	3-II, 1-IV, 1-V	*Enterococcus durans *(99%)	[GenBank:HM218637.1]
30	9, 1	5-XVIII, 1-1I	*Enterococcus faecium *(99%)	[GenBank:HQ293070.1]
	1	IV	*Lactobacillus casei *(99%)	[GenBank:HQ379174.1]
	1, 1, 2	1-4I, 1-13I, 2-XIII	*Lactobacillus plantarum *(99%)	[GenBank:EF439680.1]
31	1	1-1I	*Enterococcus *sp. (99%)	[GenBank:AB470317.1]
	1	1-3I	*Enterococcus faecium *(99%)	[GenBank:HQ293070.1]
	2, 2, 1, 2, 1, 2	2-V, 2-VII, 1-12I, 2-X, 1-4I, 2-XII	*Lactobacillus plantarum *(99%)	[GenBank:HQ441200.1]
	1	1-VIII	*Lactobacillus pentosus *(99%)	[GenBank:HM067026.1]
32	11	2-I	*Enterococcus faecium *(99%)	[GenBank:B470317.1]
	1, 1, 1	1-III, 1-15I, I-12I	*Lactobacillus casei *(99%)	[GenBank:HQ379174.1]
33	6	2-X	*Enterococcus *sp. (99%)	[GenBank:AB470317.1]
	3, 1, 1, 2	3-III, 1-VII, 1-VIII, 2-IX	*Lactobacillus plantarum *(99%)	[GenBank:HQ441200.1]

Heathy children (HC)				

34	1	1-4I^b^	*Enterococcus *sp. (99%)	[GenBank:AB470317.1]
	1	1-II	*Lactobacillus rhamnosus *(99%)	[GenBank:HM218396.1]
	2	1-IV	*Lactobacillus casei *(99%)	[GenBank:HQ379174.1]
	6	2-XI	*Lactobacillus plantarum *(99%)	[GenBank:HQ441200.1]

^a^Randomly Amplified Polymorphic DNA-Polymerase Chain Reaction (RAPD-PCR) analysis was carried out to exclude clonal relatedness. ^b^Number of cluster in Figure 4-5-6 A-B).

**Figure 4:**
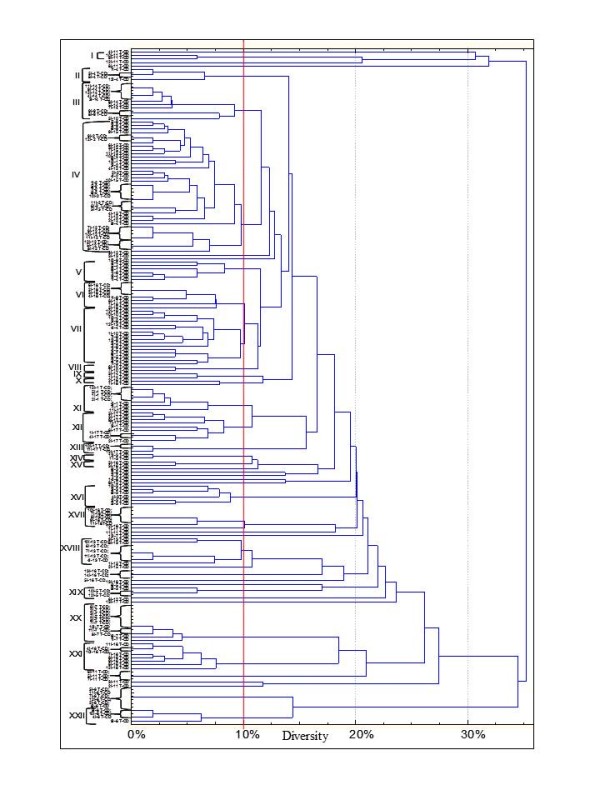
**Dendrogram of combined RAPD patterns for *Enterococcus *using primer P7, P4 and M13**. Isolates were from faecal samples of treated celiac disease (T-CD). Cluster analysis was based on the simple matching coefficient and unweighted pair grouped method, arithmetic average. *Enterococcus *and *Lactobacillus *isolates (I) are coded based on partial 16S rRNA, recA and *pheS *gene sequence comparisons and correspond to those of Table 2.

**Figure 5:**
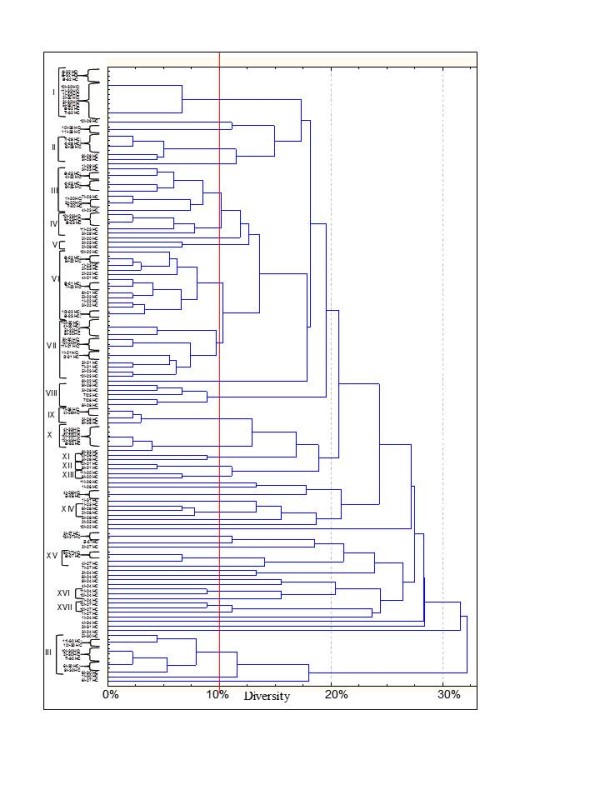
**Dendrogram of combined RAPD patterns for *Enterococcus *using primer P7, P4 and M13**. Isolates were from faecal samples of non-celiac children (HC). Cluster analysis was based on the simple matching coefficient and unweighted pair grouped method, arithmetic average. *Enterococcus *and *Lactobacillus *isolates (I) are coded based on partial 16S rRNA, *recA *and *pheS *gene sequence comparisons and correspond to those of Table 2.

**Figure 6:**
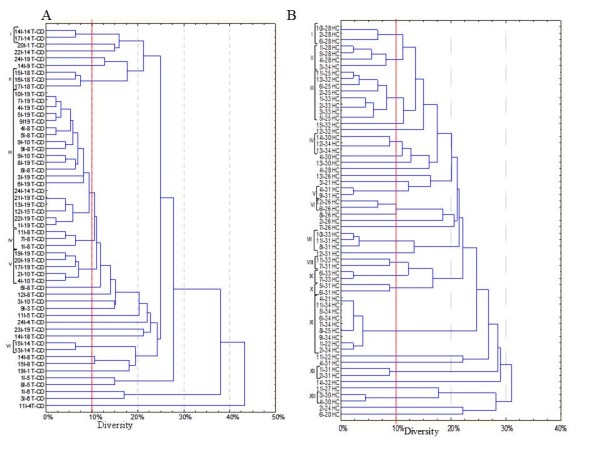
**Dendrogram of combined RAPD patterns for *Lactobacillus *using primer P7, P4 and M13**. Isolates were from faecal samples of treated celiac disease (T-CD) (A) and non-celiac children (HC) (B). Cluster analysis was based on the simple matching coefficient and unweighted pair grouped method, arithmetic average. *Enterococcus *and *Lactobacillus *isolates (I) are coded based on partial 16S rRNA, *recA *and *pheS *gene sequence comparisons and correspond to those of Table 2.

To exclude clonal relatedness and to characterize lactobacilli and enterococci, three primers (M13, P4 and P7), with arbitrarily chosen sequences, were used for RAPD-PCR analysis. The number of strains identified for each faecal sample is shown in Table 2 and Figures 4, 5 and 6A-B. The percentage of strains identified as lactobacilli significantly (*P *= 0.011) differed between T-CD (ca. 26.5%) and HC (ca. 34.6%) groups.

### Volatile organic compounds (VOC)

**Figure 7:**
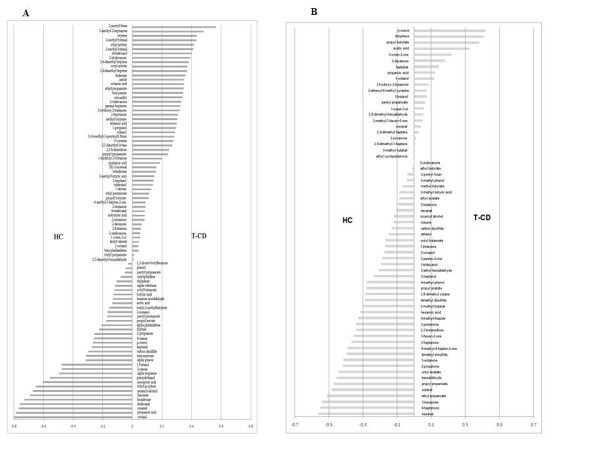
**Canonical Discriminant Analysis of Principal Coordinates (CAP) loading coefficient plot of the volatile organic metabolites from faecal (A) and urine (B) samples of treated celiac disease (T-CD) and non-celiac children (HC)**. Data are the means of three independent experiments (n = 3).

VOC (107 compounds) were identified from faecal and urine samples (Table 3 and Additional file 1, Table S1). VOC were grouped according to chemical classes: esters (14 compounds identified); sulfur compounds (3), ketones (21), hydrocarbons (15), aldehydes (16), alcohols (15), alkane (4), alkene (1), aromatic organic compounds (6), hetpane (1) and short chain fatty acids (SCFA) (11). During sampling, the level of VOC of each child did not differ (*P *> 0.05). On the contrary, high variability was found among children. Statistical differences (*P *< 0.05) were found between T-CD and HC children. As expected, faecal samples had higher level of VOC compared to urines. The median value of esters was higher than in HC children. Nevertheless, the levels of ethyl-acetate, octyl-acetate, propyl-butyrate, propyl-propanoate and butyl 2-methylbitanoate were higher than in faecal samples of T-CD. Among sulfur compounds, carbon disulfide was at higher level than in faecal samples of HC. Dimethyl trisulfide and dimethyl disulfide were at higher level than in the urine samples of HC. With a few exceptions, hydrocarbons were found at higher levels than in urine and, especially, faecal samples of HC. Faecal samples of HC contained higher median values of aldehydes compared to T-CD. The level of aldehydes did not differ (*P *> 0.05) between urine samples of T-CD and HC. Compared to faecal samples of HC, some alcohols (e.g., 1-octen-3-ol, ethanol and 1-propanol) were present at higher level in T-CD. Median values of alkane and alkene did not significantly (*P *> 0.05) differ between T-CD and HC. Overall, faecal samples of T-CD showed the lowest levels of aromatic organic compounds. The median value of total short chain fatty acids (SCFA) was significantly (*P *< 0.05) higher in faecal samples HC compared to T-CD. Major differences were found for isocaproic, butyric and propanoic acids (*P *< 0.038, 0.021, and 0.012, respectively). On the contrary, acetic acid was higher in T-CD compared to HC samples. The differences of the metabolomes between faecal or urine samples of T-CD and HC was highlighted through CAP analysis which considered only significantly different compounds (Figure 7A and 7B). Variables appearing with negative values represent bins whose values decreased in T-CD compared to HC samples. On the contrary, variables represented with bars pointing to the right indicate bins whose values were the highest in T-CD samples.

**Table 3:** Median values and ranges of the concentration (ppm) of volatile organic compounds (VOC) of faecal and urine samples from treated celiac disease (T-CD) children and non-celiac children (HC) as determined by gas-chromatography mass spectrometry/solid-phase microextraction (GC-MS/SPME) analysis

Chemical class	Treated celiac disease (T-CD)children				Non-celiac children (HC)			
	
	Faeces		Urines		Faeces		Urines	
	Median	Range	Median	Range	Median	Range	Median	Range
Esters	20.31^b^	0 - 846.97	0.47^c^	0 - 40.00	47.73^a^	1.83 - 496.83	0.99^c^	0 - 8.05
Sulfur compounds	214.83^b^	0 - 890.86	1.46^c^	0 - 25.44	387.07^a^	0 - 499.88	3.49^c^	0 - 63.67
Ketones	90.88^b^	0 - 2402.50	54.01^c^	0 - 295.03	112.83^a^	0 - 416.20	64.49^c^	0 - 458.78
Hydrocarbons	16.69^b^	0 - 1327.15	4.25^c^	0 - 67.07	119.13^a^	0.22 - 635.25	3.14^c^	0.15 - 62.56
Aldehydes	17.59^c^	0 - 512.28	64.31^a^	0.34 - 166.31	37.46^b^	2.08 - 365.25	73.37^a^	0.50 - 199.56
Alcohols	230.14^a^	0 - 2311.29	2.25^c^	0 - 17.5	122.56^b^	0 - 934.22	2.14^c^	0 - 34.96
Alkane	6.73^a^	0 - 653.61	0.3^b^	0.05 - 1.57	9.37^a^	0 - 432.74	0.43^b^	0 - 1.47
Alkene	0^a^	0 - 32.51	0^a^	0	0^a^	0 - 31.99	0^a^	0
Aromatic organic compounds	178.24^b^	0 - 143.67	2.10^c^	0.04 - 28.16	480.20^a^	233.74 - 993.94	2.78^c^	0 - 16.30
Heptane	23.01^a^	0 - 837.50	0^c^	0 - 1.37	26.37^a^	0 - 65.75	0.34^b^	0 - 2.37
Short chain fatty acids (SCFA)	21.64^a^	0 - 1438.28	3^b^	0.08 - 31.14	27.85^a^	0 - 1037.50	3.82^b^	1.44 - 24.87

Data are the means of three independent experiments (n = 3) for each children.

^a-c^Means within a row with different superscript letters are significantly different (*P *< 0.05).

### ^1^H Nuclear Magnetic Resonance (NMR) metabolite profiling of faeces and urine samples

Overall, ^1^H NMR results confirmed the trends and the major differences found between T-CD and HC samples through GC-MS/SPME analysis. Besides, other metabolites were found (Table 4). Try, Pro, Asn, His, Met, trimethylamine-N-ox and tyramine were higher in faecal samples of T-CD than HC children. By comparing the spectra of urine samples, median values of Lys, Arg, creatine and methylamine were higher than in T-CD children. On the contrary, median values of carnosine, glucose, glutamine and 3-methyl-2-oxobutanoic acid were the highest in HC children.

**Table 4:** Median values and ranges of the relative concentration (‰) of organic compounds of faecal and urine samples from treated celiac disease (T-CD) children and non-celiac children (HC) as determined by ^1^H nuclear magnetic resonance (NMR) spectroscopy analysis

Chemical class	Treated celiac disease (T-CD) children		Non-celiac children (HC)	
	**Median**	**Range**	**Median**	**Range**
Faeces				
Tryptophane	1.13^a^	0.29 - 1.38	0.68^b^	0.19 - 1.33
Proline	2.74^a^	0 - 19.68	1.87^b^	0.71 - 6.47
Trimethylamine-N-ox	3.36^a^	1.16 - 11.60	1.82^b^	0.46 - 10.94
Histidine	5.56^a^	3.05 - 19.95	2.89^b^	0.93 - 11.03
Asparagine	2.01^a^	1.02 - 2.75	1.21^b^	0.51 - 2.17
Tyramine	2.81^a^	1.34 - 3.21	1.88^b^	0.74 - 7.87
Methionine	1.78^a^	0.99 - 3.30	1.50^a^	0.64 - 2.06
Urines				
Carnosine	0.28^b^	0.12 - 0.48	0.43^a^	0.22 - 1.37
Glucose	14.66^b^	4.80 - 31.00	19.76^a^	15.33 - 53.73
Creatinine	38.51^a^	15.83 - 83.23	21.31^b^	10.40 - 61.80
Methylamine	1.45^a^	0.80 - 7.72	0.93^b^	0.32 - 2.36
Glutamine	4.05^b^	1.72 - 8.03	5.65^a^	3.14 - 8.55
Lysine-Arginine	8.96^a^	4.07 - 25.72	7.10^b^	5.59 - 11.08
Ornithine	1.87^a^	0.09 - 23.40	1.17^a^	1.03 - 2.08
3-Methyl-2-oxobutanoic acid	1.84^b^	1.12 - 2.60	2.35^a^	1.63 - 2.78

Data are the means of three independent experiments (n = 3) for each children.

^a-b^Means within a row with different superscript letters are significantly different (*P *< 0.05).

## Discussion

This study used culture-independent and culture-dependent methods and metabolomics analyses to investigate the differences in the microbiota and metabolome of 19 treated celiac disease (T-CD, under remission since 2 years) children and 15 non-celiac children (HC). The present study showed that the whole eubacterial community significantly changed between the duodenal microbiota of T-CD and HC children. In agreement, other authors [9] reported similar results when faecal samples of CD children were compared to those of HC. This result was surprising since an heterogeneous group like the 'healthy controls' should have more heterogeneity in DGGE microbial profiles. However, also Schippa et al [26] showed a peculiar microbial TTGE profile and a significant higher biodiversity in CD pediatric patients' duodenal mucosa after 9 months of GFD compared to healthy control. As determined by PCR-DGGE analysis, the population of lactobacilli from duodenal biopsies of children was relatively simple and homogeneous, having only one dominant bacterial band which corresponded to *L. plantarum*. Under the experimental conditions of this study, bifidobacteria were not detected on duodenal biopsies of T-CD and HC children. Recently, it was shown that bifidobacteria were present at high levels in duodenal biopsies of CD children at diagnosis and they decreased in T-CD and, especially, in HC [27]. *Bifidobacterium *species were demonstrated to have species- and strain-specific influence on immunity, and they might exert various effects on T-helper 1 pro-inflammatory response which characterizes CD [17]. Nevertheless, the association between the prevalence of *Bifidobacterium *species and CD is still debated [27].

Compared to duodenal biopsies, the microbial diversity was larger in faecal samples. If some bands seem to be clearly present only in HC or T-CD duodenal biopsies, on the other hand, this is not so evident in faecal samples very likely because of the high number of bands quite different among all samples. With a few exceptions, PCR-DGGE profiles of *Lactobacillus *and *Bifidobacterium *differed between faecal samples of T-CD and HC children. Overall, the faecal bacterial population is significantly affected by individuals, diet and CD [9,10,20,21,27].

As determined by culture-dependent methods, cell densities of the main faecal microbial groups differed between T-CD and HC children. In agreement with the previous report [10], the ratio between lactic acid bacteria-*Bifidobacterium *and *Bacteroides*-*Enterobacteria *was lower in T-CD compared to HC children. Increased numbers of *Bacteroides *are usually found in faecal samples of children affected by GI inflammatory diseases, including CD [13,16]. In the present study, lactic acid bacteria were identified and subjected to RAPD-PCR analysis for determining qualitative and quantitative differences between T-CD and HC. *E. faecium *was the dominant species of both T-CD and HC children. *L. plantarum*, *L. casei *and *L. rhamnosus *were found on faecal samples of both T-CD and HC. *Str. macedonicus*, *Str. pasterianus*, *P. pentosaceus *and *P. acedilactici *were only isolated from T-CD. Although the RAPD-PCR and 16S rRNA gene analyses were successfully applied in this study as well as in others [10,28], more performing techniques (e.g., species and/or strain specific probes for real time PCR or end-point PCR) [29,30], would be desirable for a rapid enumeration of live lactic acid bacteria in the human microbiota. Contrarily to the previous study [10], *L. fermentum *and *L. delbrueckii *subsp. *bulgaricus *were only isolated from faecal samples of T-CD. Recently, it was shown that the prevalence of amplicons of the species *L. fermentum *was higher in CD compared to HC children [27]. Since lactobacilli are routinely present in fermented foods, some of the differences found in this study could be related to CD, but also to dietary differences [27]. As showed by RAPD-PCR analysis, the percentage of isolation of *Lactobacillus *strains was the lowest in T-CD which agreed with other reports [10,27].

The qualitative and quantitative differences found for GI microbiota affected the level of volatile organic compounds (VOC) and amino acids in faecal and urine samples. A few studies considered the metabolome of faecal or urine samples [10,22]. The concept of human metabolome encompasses the idea of microbial and metabolic cooperation, and it aims to systematically examine changes in numerous low molecular mass metabolites of biological fluids as the response to different stimuli such as drugs or diseases [31-33]. The combination of GC-MS/SPME and ^1^H NMR metabolic profiles together with CAP analysis allowed the identification of specific molecules which significantly changes in T-CD children. The largest level of esters was found for HC, whereas ethyl-acetate and octyl-acetate seemed to be over-synthesized in T-CD children. Overall, esterification reactions at the colon level are considered as the microbial strategy to remove or detoxify acids or alcohols [34]. Median values of aldehydes were the highest in HC compared to T-CD children. Previously, the highest level of alcohols was found in CD children at diagnosis compared to T-CD and HC [10]. In this study, some alcohols such as 1-octen-3-ol, ethanol and 1-propanol were higher in T-CD than HC children. Ethanol seems to be an important mediator to develop of non-alcoholic steatohepatitis (NASH). It was hypothesized that when intestinal bacteria synthesize alcohol they may induce endotoxemia [35]. NASH was also associated to occult CD [36]. The present study confirmed the higher level of some short chain fatty acids (SCFA) of HC compared to T-CD children [10,37]. It was suggested that *Lactobacillus *and *Bifidobacterium *modified the metabolism of the large intestine by increasing the synthesis of SCFA [10,38]. SCFA are some of the most important by-products of anaerobes in the colon. They represent the main fuel for colonocytes and are involved in water and electrolyte absorption by colon mucosa, even under diarrheic conditions [39]. The increase of butyric acid is especially significant since it plays a key role in the regulation of cell proliferation and differentiation of colon epithelial cells. It was also shown that faecal and urine samples of T-CD had an altered level of free amino acids compared to HC children. Indeed, a large number of free amino acids and related compounds were found at the highest level in T-CD children. Another report [22], also showed that serum and urine samples of adult CD patients had altered level of amino acids. Peptides enter enterocytes either after preliminary digestion by brush border peptidases into amino acids or as di- and tri-peptides which are split inside the cell by cytoplasmic peptidases. Non specific inflammatory alterations of the intestinal mucosa (e.g., CD), which are associated with a significant decrease of the absorptive surface and brush border enzyme activity, may cause the decrease of amino acid/peptide absorption which are consequently lost with stools [40]. Dietary amino acids are the major fuel for the small intestinal mucosa as well as they are important substrates for the synthesis of intestinal proteins such as nitric oxide polyamines and other products with enormous biological activity [41]. Glutamine was one of the few free amino acid related compounds which was found at the highest level in HC children. A low level of glutamine was also previously found in CD children and adults [22]. Specific amino acids and related compounds, including glutamine, were shown to possess a therapeutic role in gut diseases [41]. This study confirmed the hypothesis that CD is associated with intestinal and faecal dysbiosis, which is related to certain bacterial species. Recently, it was shown that potential celiac subjects and overt celiac subjects show differences in the urine metabolites and a very similar serum metabolic profile [42]. Metabolic alterations may precede the development of small intestinal villous atrophy and provide a further rationale for early institution of GFD in patients with potential CD [42]. As shown by both microbiology and metabolome analyses, the GFD lasting at least two years did not completely restore the microbiota and, consequently, the metabolome of CD children. Probably, the addition of prebiotics and probiotics to GFD might restore the balance of microbiota and metabolome of CD children.

## Conclusions

As shown by the microbiology and metabolome studies, the gluten-free diet lasting at least two years did not completely restore the microbiota and, consequently, the metabolome of CD children. Combining the results of this work with those from previous reports [9,10,16,22,27,37], it seems emerge that microbial indeces (e.g., ratio between faecal cell density of lactic acid bacteria-*Bifidobacterium *vs. *Bacteroides*-*Enterobacteria*) and levels of some metabolites (e.g., ethyl-acetate, octyl-acetate, SCFA and glutamine) are signatures of CD patients. Further studies, using a major number of children and a complete characterization of all microbial groups, are in progress to find a statistical correlation between the microbiota and metabolome of T-CD compared to HC children.

## Methods

### Subjects

Two groups of children (6 - 12 years of age) (Table 5) were included in the study: (i) nine-teen symptom-free CD patients, who had been on a GFD for at least 2 years (treated CD children, T-CD) (children numbered: 1 - 19 T-CD); and (ii) fifteen children without celiac disease and other known food intolerance undergoing upper endoscopy for symptoms related to functional dyspepsia and in whom endoscopy showed no signs of disease (non-celiac children) (children numbered: 20 - 34 HC). The pathology was diagnosed according to criteria given by the European Society for Pediatric Gastroenterology, Hepatology, and Nutrition. Children included in the study were not treated with antibiotics and/or functional foods (probiotics and/or prebiotics) for three months before sampling. Children were enrolled in the study after written informed consent, that was obtained both from the respective parents and the institutional ethics committee of the Faculty of Medicine and Surgery of the University of Bari Aldo Moro, Italy.

**Table 5:** Demographic and clinical characteristic of the children included in the trial

	Age Median (range)	F/M	Cesarean section	Feeding habits	IEC* Median (range)	Marsh score*
Celiac children	9.7 (6 - 12) years	11/8	68%	Strict gluten free diet	34 (26-50)	3c
Non-celiac children	10.4 (6 - 12) years	8/7	60%	Unrestricted	5 (0-12)	0

*At diagnosis

### Collection of duodenal biopsies, faecal and urine samples

Each child had fasted overnight, and biopsies, which were taken always from the second duodenum, faecal and urine were collected in the morning pre-prandial. Urine samples were collected after the second mittus. Each child provided a duodenal biopsy and three faecal and urine samples over the time. Duodenal biopsy specimens were obtained from the second duodenum by upper intestinal endoscopy, frozen immediately at -80°C and kept until further processing. After collection, faeces (ca. 15 g), contained in sterile plastic box, were immediately mixed (1:1 wt/wt) with the Amies Transport medium (Oxoid LTD, Basingstoke, Hampshire, England) under anaerobic conditions (AnaeroGen, Oxoid LTD). Samples were immediately subjected to analysis (plate counts) or frozen at -80°C (DNA extraction). The urine samples were collected into pre-labeled sterile collections cups. Three aliquots per patient were immediately frozen and stored at -80°C until use.

### DNA extraction from duodenal biopsies and faecal samples

Biopsies specimens, the average weight was ca. 3.5 mg (biopsies are not usually weighted, however all were taken by the same endoscopist using the same biopsy forceps), were homogenized using a sterile plastic pestle in 200 μl of 20 mM Tris-HCl, pH 8.0, 2 mM EDTA buffer. The homogenate was subjected to mechanical disruption in a FastPrep^® ^instrument (BIO 101) and total DNA was extracted with a FastDNA^® ^Pro Soil-Direct Kit (MP Biomedicals, CA., USA) according to the manufacturer's instructions. Three samples of faecal slurry of each child were mixed and used for DGGE analysis [43]. An aliquot of about 300 μl of each faecal slurry sample containing 150 μg of faeces was diluted in 1 ml of PBS-EDTA (phosphate buffer 0.01 M, pH 7.2, 0.01 M EDTA). After centrifugation (14,000 × g at 4°C for 5 min), the pellet was washed two times to decrease the content of PCR inhibitors. The resulting pellet was resuspended in 300 μl of PBS-EDTA and used for DNA extraction [44] with a FastPrep instrument as above. The final product was 100 μl of application-ready DNA both for stool and tissue samples [45]. Quality and concentration of DNA extracts were determined in 0.7% agarose-0.5X TBE gels stained with Gel Red ™ 10,000X (Biotium, Inc.) and by spectrophotometric measurements at 260, 280 and 230 nm using the NanoDrop^® ^ND-1000 Spectrophotometer (ThermoFisher Scientific Inc., MI., Italy).

### Polymerase chain reaction (PCR) amplification and denaturing gel electrophoresis (DGGE) analysis

DNA isolated from duodenal biopsy and faecal samples was subsequently used as the template in PCR assays applying eubacterial universal and group-specific 16S rRNA gene primer sets. All primers used in this study are listed in Table 1. The forward or the reverse primer of each set was extended with a 40 mer GC-clamp at the 5' end to separate the corresponding PCR products in the gradient gel [46]. The specificity of each primer pair was experimentally tested by using DNA extracted from the following bacteria species: *Bacteroides fragilis *DSM 2151*, Bifidobacterium bifidum *DSM 20082*, L. plantarum *ATCC 14917*, Weissella confusa *DSM2196*, P. pentosoceus *DSM 20336*, Leuconostoc lactis *DSM 20202*, E. durans *DSM 20633*, E. faecium *DSM 2918*, Clostridium coccoides *DSM 935, *Staphylococcus aureus *DSM 20714, *Enterobacter aerogenes *DSM 30053, *Escherichia coli *DSM 30083 and *Yersinia enterocolitica *DSM 4780. Each primer set gave positive PCR results for the corresponding target bacteria and did not cross-react with any of the non target microorganisms. Each PCR mixture contained 80 - 100 ng and 40 ng of template DNA extracted from bioptic materials and faecal samples respectively, 50 pmol of each primer, 10 nmol of each 2'-deoxynucleoside 5'-triphosphate (dNTP), 3 U of Taq DNA polymerase (EuroTaq, EuroClone, Italy) and 2.5 mM MgCl_2 _in a buffered final volume of 50 μl. The following PCR core program was used for the first three primer pairs listed in Table 1: initial denaturation at 95°C for 3 min; 30 cycles of denaturation at 95°C for 20 s, annealing at primer-specific temperature for 45 s and extension at 72°C for 1 min; and final extension at 72°C for 7 min. DNA extracted from duodenal biopsies was amplified by two additional set of primers targeting *Bifidobacterium *group and the PCR thermocycling program used for both Bif164-f/Bif662-GC-r and Bif164-GC-f/Bif662-r was: 94°C for 5 min; 35 cycles of 94°C for 30 s, 62°C for 20 s, and 68°C for 40 s; and 68°C for 7 min [47]. PCR amplification products were checked by electrophoresis in 1.5% agarose Gel Red 10,000X stained gels and stored at -20°C. Amplicons were separated by DGGE, using the Bio-Rad DCode™ Universal Mutation detection System (Bio-Rad Laboratories, Hercules, CA, USA). Different linear denaturing gradients of urea and formamide were applied depending on the amplified target sequence and type of samples (Table 1). The denaturing gradient conditions proposed by Vanhoutte et al. [43] were modified as described below. For eubacterial amplicons the denaturing gradient was 45-55% for faecal samples and 40-65% for duodenal biopsies, respectively; Lac1-Lac2GC PCR products relative to faecal and biopsies samples were separated in 35-50% and 35-70% denaturing gradient, respectively and, finally, g-BifidF/gBifidR-GC amplicons from faecal samples were resolved by 45-60% gradient. Gels were electrophoresed at 60°C at 75 V for 15 h. Sybr Green I stained gels were photographed and acquired by the Bio-Rad Gel Doc 2000 documentation system (Bio-Rad Laboratories). To compensate for internal distortions occurring during the electrophoresis, binding patterns were digitally aligned using the Bionumerics software version 4.5 (Applied Maths, Belgium) by comparison with an external reference pattern obtained by appropriately mixing DGGE marker II, III and V (Nippon gene, Tokyo), depending on the gradient used. This normalization enabled comparison among DGGE profiles from different gels, provided that these were run under comparable denaturing and electrophoretic conditions. Comparison and cluster of profiles were carried out using the unweigthed pair-group method with the arithmetic average (UPGMA) clustering algorithm based on the Pearson product-moment correlation coefficient (r) [25,48] and resulted in a distance matrix. DGGE fragments from primers Lac1 and Lac2 were cut out using sterile scalpel. The DNA of each band was eluted in 100 μl of sterile water overnight at 4°C. Two μl of the eluted DNA were reamplified as described above. PCR products were separated by electrophoresis on 1.5% (wt/vol) agarose gel (Gibco BRL, France) stained with ethidium bromide (0.5 μg/ml). The amplicons were eluted from gel and purified by the GFXTM PCR DNA and Gel Band Purification Kit (GE Healthcare Life Sciences, Milan, Italy). DNA sequencing reactions were performed by MWG Biotech AG (Ebersberg, Germany). Sequences were compared to the GenBank database with the BLAST program.

### Enumeration of cultivable bacteria

Diluted faecal samples (20 g) were mixed with 80 ml sterilized peptone water and homogenized. Counts of viable bacterial cell were carried out as described by Macfarlane et al. [45,49] The following selective media were used: MRS agar (lactobacilli); Beerens agar (bifidobacteria); Baird-Parker (staphylococci and micrococci); Blood Azide agar (enterococci); Wilkins-Chalgren agar (total anaerobes); Wilkins-Chalgren agar plus GN selective supplements (*Bacteroides*, *Porphyromonas *and *Prevotella*); Reinforced Clostridial Medium supplemented with 8 mg/l novobiocin, 8 mg/l colistin (*Clostridium*), MacConkey agar No2 (enterobacteria); and nutrient agar (total anaerobes) [50].

### Lactic acid bacteria isolation

Fifteen to twenty colonies of presumptive lactic acid bacteria were isolated from the highest plate dilutions of MRS and Blood Azide agar media. Gram-positive, catalase-negative, non-motile rods and cocci isolates were cultivated in MRS or Blood Azide broth (Oxoid Ltd) at 30, 37 or 42°C for 24 h, and re-streaked into the same agar media. All isolates considered for further analyses showed the capacity of acidifying the liquid culture medium. All cultures were stored at -80°C in 10% (vol/vol) glycerol.

### DNA extraction and molecular identification by 16S rRNA, pheS and recA genes sequencing

Total DNA of presumptive lactic acid bacteria isolates was extracted from 2 ml samples of overnight cultures grown at 37°C in MRS or Blood Azide broth. Total DNAs were obtained as described by De Los Reyes-Gavilàn et al. [51]. The concentration and purity of DNA was assessed by a NanoDrop^® ^ND-1000 Spectrophotometer (Thermo Fisher Scientific Inc.). A primer pair (Invitrogen Life Technologies, Milan, Italy), LpigF/LpigR (5'-TACGGGAGGCAGCAGTAG-3' and 5'-CATGGTGTGACGGGCGGT-3') [52], corresponding to the position 369-386, and 1424-1441, respectively, of the 16S rRNA gene sequence of *L. mucosae*, (accession number AF126738) was used to amplify the 16S rRNA gene fragment of presumptive lactic acid bacteria. Fifty microliters of each PCR mixture contained 200 μM of each dNTP, 1 μM of both forward and reverse primer, 2 mM MgCl_2_, 2 U of *Taq *DNA polymerase (Invitrogen Life Technologies) in the supplied buffer, and approximately 50 ng of DNA. PCR amplification was carried out using the GeneAmp PCR System 9700 thermal cycler (Applied Biosystems, USA). PCR products were separated by electrophoresis on 1.5% (wt/vol) agarose gel (Gibco BRL, France) stained with ethidium bromide (0.5 mg/ml). The amplicons were eluted from gel and purified by the GFX™ PCR DNA and Gel Band Purification Kit (GE Healthcare Life Sciences, Milan, Italy). DNA sequencing reactions were carried out by MWG Biotech AG (Ebersberg, Germany) using both, forward and reverse, primers. Taxonomic identification of strains was performed by comparing the sequences of each isolate with those reported in the Basic BLAST database http://www.ncbi.nlm.nih.gov. Primers casei/para were used to discriminate between the species *L. casei*, *L. paracasei *and *L. rhamnosus *[53]. Primers pheS-21-F/pheS-23-R were used to identify *Enterococcus *species [54]. Primers designed on *recA *gene were also used to discriminate between the species *L. plantarum*, *L. pentosus *and *L. paraplantarum*. Part of the *recA *gene was amplified using the degenerate primer pair (MWG Biotech AG, Ebersberg, Germany) recALb1F 5'-CRRTBATGCGBATGGGYG-3'/recALb1R 5'-CGRCCYTGWCCAATSCGRTC-3' derived from the homologous regions of the *recA *gene sequences of *L. plantarum *(accession no. AJ621668). PCR reactions and separation, and purification and sequencing of amplicons were carried out as described for 16S rRNA gene.

### Genotypic characterization by Randomly Amplified Polymorphic DNA-Polymerase Chain Reaction (RAPD-PCR) analysis

Genomic DNA from each isolates was extracted as described above. Three oligonucleotides, P4 5'-CCGCAGCGTT-3', P7 5'-AGCAGCGTGG-3' and M13 5'-GAGGGTGGCGGTTCT-3' [55,56], with arbitrarily chosen sequences, were used for isolates biotyping. Reaction mixture and PCR conditions for primers P4 and P7, and primer M13 were according to De Angelis et al. [55,56]. PCR products (15 μl) were separated by electrophoresis at 100 V for 200 min on 1.5% (wt/vol) agarose gel and DNA was detected by UV transillumination after staining with ethidium bromide (0.5 μg/ml). Molecular sizes of the amplified DNA fragments were estimated by comparison with 1-kb DNA molecular size markers (Invitrogen Life Technologies). RAPD-PCR profiles were acquired by Gel Doc EQ System (Bio-Rad Laboratories) and compared using Fingerprinting II Informatix™ Software (Bio-Rad). The similarity of the electrophoretic profiles was evaluated by determining the Dice coefficients of similarity and using the UPGMA method.

### Gas-chromatography mass spectrometry/solid-phase microextraction (GC-MS/SPME) analysis

After preconditioning according to the manufacturer's instructions, the carboxen-polydimethylsiloxane coated fiber (85 μm) and the manual SPME holder (Supelco Inc., Bellefonte, PA, USA) were used. Before head space sampling, the fiber was exposed to GC inlet for 5 min for thermal desorption at 250°C. Three grams of faecal sample were placed into 10 ml glass vials and added of 10 μl of 4-methyl-2-pentanol (final concentration of 4 mg/l), as the internal standard. Samples were then equilibrated for 10 min at 45°C. SPME fiber was exposed to each sample for 40 min. Both phases of equilibration and absorption were carried out under stirring condition. The fiber was then inserted into the injection port of the GC for 5 min of sample desorption. GC-MS analyses were carried out on an Agilent 7890A gas-chromatograph (Agilent Technologies, Palo Alto, CA, USA) coupled to an Agilent 5975C mass selective detector operating in electron impact mode (ionization voltage 70 eV). A Supelcowax 10 capillary column (60 m length, 0.32 mm ID) was used (Supelco, Bellefonte, PA, USA). The temperature program was: 50°C for 1 min, 4.5°C/min to 65°C and 10°C/min to 230°C, which was held for 25 min. Injector, interface and ion source temperatures were 250, 250 and 230°C, respectively. The mass-to-charge ratio interval was 30-350 a.m.u. at 2.9 scans per second. Injections were carried out in splitless mode and helium (1 ml/min) was used as the carrier gas. Sodium 3-(trimethylsilyl)propionate-2,2,3,3-d4 (TSP) was used as the internal standard. Identification of molecules was carried out based on comparison of their retention times with those of pure compounds (Sigma-Aldrich, Milan, Italy). Identification was confirmed by searching mass spectra in the available databases (NIST version 2005 and Wiley Vers. 1996) and literature [57]. Quantitative data of the identified compounds were obtained by interpolation of the relative areas versus the internal standard area [33].

### ^1^H Nuclear Magnetic Resonance (NMR) spectroscopy analysis

To study the water soluble fraction of the faeces by means of ^1^H NMR spectroscopy, 40 mg of thawed faecal or urine mass were thoroughly homogenized by vortex-mixing with 400 μl of cold deuterium oxide (D_2_O) at pH 7.4 ± 0.02, containing 1 mM TSP as the internal standard. Mixtures were centrifuged at 14,000 rpm for 5 min and the supernatant was collected. To ensure the complete recovery of the water soluble species and highly reproducible spectra, the extraction procedure was repeated two times [31]. ^1^H NMR spectra were acquired on the collected supernatants, with no further treatments, at 300 K on a Mercury-plus NMR spectrometer from Varian, operating at a proton frequency of 400 MHz. Residual water signal was suppressed by means of presaturation. ^1^H NMR spectra were processed by means of VNMRJ 6.1 software from Varian. To minimize the signals overlap in crowded regions, all free induction decays (FID) were multiplied by an exponential function equivalent to a -0.5 line-broadening factor and by a gaussian function with a factor of 1. After manual adjustments of phase and baseline, the spectra were scaled to the same total area, in order to compare the results from samples of different weight and water and fiber content. The spectra were referenced to the TSP peak, then digitized over the range of 0.5 - 10 ppm. By means of R scripts developed in-house the residual water signal region, 4.5 - 5.5 ppm, was excluded from the following computations [58]. To compensate for chemical-shift perturbations, the remaining original data points were reduced to 218 by integrating the spectra over 'bins', spectral areas with a uniform size of 0.036 ppm. A 34 × 218 bins table was thus obtained for statistical analysis. As some parts of the spectra are very crowded, some bins may contain peaks pertaining to different molecules. In order to consider this potential source of error the bins containing peaks ascribed to the same molecules were not summed up [33].

### Statistical analysis

All data coming from culture-dependent analysis and metabolomic analysis were obtained at least in triplicates. The analysis of variance (ANOVA) on culture-dependent analysis, GC-MS/SPME and ^1^H-NMR analysis, was carried out on transformed data followed by separation of means with Tukey's HSD, using a statistical software Statistica for Windows (Statistica 6.0 per Windows 1998, (StatSoft, Vigonza, Italia). Letters indicate significant different groups (*P *< 0.05) by Tukey's test. Canonical discriminant Analysis of Principal coordinates (CAP) analysis was carried out for GC-MS/SPME data [33]. This was preferred to the more common Canonical Discriminant Analysis (CDA), because it does not assume any specific distribution of the data, thus giving more robust results in the case of reduced number of samples. The CAP constrained ordination procedure that was carried out is summarized as follows: (i) data were reduced by performing a Principal Coordinate analysis (PCO) of the parameters, using the dissimilarity measure calculated on euclidean distances; (ii) an appropriate number of PCO was chosen non-arbitrarily, which maximizes the number of observations correctly classified; (iii) the power of classification was tested through a leave-one-out procedure; and (iv), finally, a traditional canonical analysis on the first PCO was carried out. The total variance obtained in PCO used to perform CAP was 70 and 73% for faeces and urine data, respectively. The hypothesis of no significant difference in the multivariate location within groups was tested using the trace statistic based on 9999 permutations [33]. The permutation test performed correctly assigns ca. 90% of the samples.

## Competing interests

The authors declare that they have no competing interests.

## Authors' contributions

RDC carried out the culture-dependent analyses and participated to culture-independent analyses and the discussion of results. MDA participated in the conception of the study, its design and coordination, drafted and revised the manuscript. IDP participated in the culture-independent and -dependent analyses. MN carried out the statistical analysis on metabolomic data and participated in the discussion of related results. PV carried out the GC-MS/SPME analysis and participated in the discussion of the metabolomic data. PR carried out the culture-independent analyses. FG participated to the faecal and urine collection and patients' data. LL carried out the ^1^H-NMR analysis. CC participated in design and coordination of the culture-independent analyses and helped the revision of the manuscript. MEG participated in the design of the metabolomic study and discussion of results and helped to draft the manuscript. MG participated in the conception of the study and revision of the manuscript and gave final approval to the study. RF participated in the conception of the study, coordinated and performed the study with children, collected the samples, participated to the discussion of results and helped to the revision of manuscript. All authors read and approved the final manuscript.

## Supplementary Material

Additional file 1 **Table S1: Concentration (ppm) of volatile organic compounds (VOC) of faecal and urine samples as determined by gas-chromatography mass spectrometry/solid-phase microextraction (GC-MS/SPME) analysis**. Click here for file
