# Supplementary material for: Duodenal and faecal microbiota of celiac children: molecular, phenotype and metabolome characterization
Source: BMC Microbiol. 2011 Oct 4;11:219. doi: 10.1186/1471-2180-11-219 (PMC3206437; doi:10.1186/1471-2180-11-219)
Supplement: Additional file 1 — Table S1: Concentration (ppm) of volatile organic compounds (VOC) of faecal and urine samples as determined by gas-chromatography mass spectrometry/solid-phase microextraction (GC-MS/SPME) analysis. [file 1471-2180-11-219-S1.DOC]

**Table 1S: Concentration (ppm) of volatile organic compounds (VOCs) of faecal and urine samples as determined by gas-chromatography mass spectrometry/solid-phase microextraction (GC-MS/SPME) analysis**

| **Chemical class** | **Treated coeliac children (T-CD)** | | | | **Healthy children (HC)** | | | |
| --- | --- | --- | --- | --- | --- | --- | --- | --- |
|  | **Faeces** | | **Urines** | | **Faeces** | | **Urines** | |
|  | Median | Range | Median | Range | Median | Range | Median | Range |
| Esters |  |  |  |  |  |  |  |  |
| Ethyl butyrate | 0^b^ | 0 | 0.04^a^ | 0 - 0.34 | 0^b^ | 0 | 0.04^a^ | 0 - 0.43 |
| Pentyl pentanoate | 0.06^b^ | 0 - 34.75 | 0^b^ | 0 | 15.95^a^ | 1.83 - 110.75 | 0^b^ | 0 |
| Ethyl-acetate | 4.46^a^ | 0 - 92.64 | 0.18^c^ | 0 - 0.63 | 3.09^b^ | 0 - 12.27 | 0.16^c^ | 0 - 2.01 |
| Octyl acetate | 1.31^a^ | 0 - 10.43 | 0^b^ | 0 - 0.15 | 0^b^ | 0 - 3.29 | 0.04^b^ | 0 - 0.69 |
| Ethyl pentanoate | 0^a^ | 0 - 10.82 | 0^a^ | 0 | 0^a^ | 0 - 6.15 | 0^a^ | 0 |
| Ethyl-propanoate | 1.36^b^ | 0 - 79.37 | 0^c^ | 0 - 0.24 | 4.61^a^ | 0 - 14.28 | 0.17^c^ | 0 - 1.15 |
| Propyl-acetate | 0^a^ | 0 - 9.56 | 0^a^ | 0 - 0.15 | 0^a^ | 0 - 5.06 | 0^a^ | 0 - 0.25 |
| Methyl butyrate | 2.16^b^ | 0 - 145.14 | 0^c^ | 0 - 0.45 | 3.83^a^ | 0 - 28.87 | 0^c^ | 0 - 0.33 |
| Pentyl propanoate | 0^c^ | 0 - 43.51 | 0.11^b^ | 0 - 1.52 | 14.02^a^ | 0 - 94.71 | 0.14^b^ | 0 - 0.85 |
| Propyl-butyrate | 4.09^a^ | 0 - 103.44 | 0.06^c^ | 0 - 35.41 | 2.07^b^ | 0 - 136.73 | 0.13^c^ | 0 - 0.37 |
| Butyl-propanoate | 0^b^ | 0 - 90.98 | 0^b^ | 0 | 0.43^a^ | 0 - 19.99 | 0^b^ | 0 |
| Propyl-propanoate | 2.81^a^ | 0 - 177.64 | 0.03^c^ | 0 - 0.67 | 0^c^ | 0 - 27.19 | 0.24^b^ | 0 - 1.13 |
| Octyl butanoate | 2.85^b^ | 0 - 19.31 | 0.06^c^ | 0 - 0.44 | 3.73^a^ | 0 - 19.84 | 0.07^c^ | 0 - 0.84 |
| Butyl 2-methylbutanoate | 1.21^a^ | 0 - 29.38 | 0^b^ | 0 | 0^b^ | 0 - 17.70 | 0^b^ | 0 |
| Total esters | 20.31^b^ |  | 0.47^c^ |  | 47.73^a^ |  | 0.99^c^ |  |

**Table 1S** continued

| **Chemical class** | **Treated coeliac children (T-CD)** | | | | **Healthy children (HC)** | | | |
| --- | --- | --- | --- | --- | --- | --- | --- | --- |
|  | **Faeces** | | **Urines** | | **Faeces** | | **Urines** | |
|  | Median | Range | Median | Range | Median | Range | Median | Range |
| Sulfur compounds |  |  |  |  |  |  |  |  |
| Carbon Disulfide | 214.83^b^ | 0 - 890.86 | 0.50^c^ | 0 - 1.49 | 387.07^a^ | 173.66 - 499.88 | 0.40^c^ | 0 - 6.65 |
| Dimethyl trisulfide | 0^c^ | 0 | 0.46^b^ | 0 - 18.57 | 0^c^ | 0 | 2.4^a^ | 0 - 53.61 |
| Dimethyl disulfide | 0^c^ | 0 | 0.50^b^ | 0 - 5.38 | 0^c^ | 0 | 0.64^a^ | 0 - 3.41 |
| Total sulfur compounds | 214.83^b^ |  | 1.46^c^ |  | 387.07^a^ |  | 3.49^c^ |  |
| Ketones |  |  |  |  |  |  |  |  |
| Pentan-2-one | 0^b^ | 0 | 0.31^a^ | 0 - 0.83 | 0^b^ | 0 | 0.33^a^ | 0.08 - 0.97 |
| 3-Penten-2-one | 0^b^ | 0 | 0.17^a^ | 0.05 - 0.95 | 0^b^ | 0 | 0.18^a^ | 0 - 0.72 |
| 3-Hexen-2-one | 0^c^ | 0 | 0.25^b^ | 0 - 1.78 | 0^c^ | 0 | 0.50^a^ | 0.06 - 3.52 |
| 2-Tridecanone | 0^b^ | 0 - 16.65 | 0^b^ | 0 | 2.05^a^ | 0 - 10.65 | 0^b^ | 0 |
| 6-Methyl-5-hepten-2-one | 13.28^a^ | 0 - 178.31 | 0.456^b^ | 0.11 - 1.78 | 16.88^a^ | 0 - 31.83 | 0.70^b^ | 0.11 - 1.21 |
| 2-Undecanone | 0^a^ | 0 - 5.65 | 0^a^ | 0 - 0.23 | 0^a^ | 0 - 0.43 | 0^a^ | 0 - 0.24 |
| 2-Decanone | 2.41^b^ | 0 - 23.07 | 0^c^ | 0 - 0.52 | 3.02^a^ | 0 - 14.62 | 0^c^ | 0 - 0.30 |
| 2-Nonanone | 1.91^a^ | 0 - 60.48 | 0^c^ | 0 - 8.01 | 0.49^b^ | 0 - 10.22 | 0^c^ | 0 - 0.05 |
| 1-Hydroxy-2-butanone | 0.56^b^ | 0 - 11.58 | 0^c^ | 0 | 0.95^a^ | 0 - 3.07 | 0^c^ | 0 |
| 2-Propanone | 0^c^ | 0 - 32.93 | 2.77^b^ | 0 - 145.64 | 0^c^ | 0 - 58.74 | 3.56^a^ | 0 - 203.46 |
| 2-Butanone | 14.49^a^ | 0 - 136.19 | 1.96^b^ | 0 - 9.84 | 13.54^a^ | 2.23 - 42.09 | 2.76^b^ | 0.30 - 4.76 |
| 4-Heptanone | 0^c^ | 0 | 23.16^b^ | 0 - 65.58 | 0^c^ | 0 | 32.13^a^ | 1.63 - 154.57 |

**Table 1S** continued

| **Chemical class** | **Treated coeliac children (T-CD)** | | | | **Healthy children (HC)** | | | |
| --- | --- | --- | --- | --- | --- | --- | --- | --- |
|  | **Faeces** | | **Urines** | | **Faeces** | | **Urines** | |
|  | Median | Range | Median | Range | Median | Range | Median | Range |
| 2-Heptanone | 4.09^a^ | 0 - 164.13 | 2.18^b^ | 0 - 5.97 | 2.22^b^ | 0.71 - 7.61 | 1.94^c^ | 0 - 10.61 |
| 2-Hexanone | 0^b^ | 0 - 347.96 | 0^b^ | 0 | 2.14^a^ | 0 - 21.25 | 0^b^ | 0 |
| 3-Hexanone | 0^b^ | 0 | 0.26^a^ | 0 - 1.11 | 0^b^ | 0 | 0.40^a^ | 0 - 1.42 |
| Ethyl cyclopentanone | 0^c^ | 0 | 0.46^b^ | 0.11 - 1.78 | 0^c^ | 0 | 1.50^a^ | 0.10 - 9.61 |
| 2,3-Butanedione | 39.03^a^ | 0 - 400.54 | 4.95^c^ | 0 - 15.68 | 30.46^a^ | 10.14 - 89.21 | 9.85^b^ | 0 - 34.54 |
| 3-Octen-2-one | 0^c^ | 0 | 0.22^a^ | 0 - 1.04 | 0^c^ | 0 | 0.08^b^ | 0 - 0.43 |
| 3-Octanone | 0^b^ | 0 | 0.02^a^ | 0 - 0.99 | 0^b^ | 0 | 0.02^a^ | 0 - 1.80 |
| 2-Methyl-3-decen-5-one | 0^c^ | 0 | 16.60^a^ | 0.21 - 29.79 | 0^c^ | 0 | 10.34^b^ | 4.08 - 28.50 |
| 3-Hydroxy-2-butanone | 15.11^b^ | 0 - 1025.01 | 0.23^c^ | 0 - 3.51 | 41.08^a^ | 0 - 126.48 | 0.26^c^ | 0 - 2.07 |
| Total Ketones | 90.88^b^ |  | 54.01^c^ |  | 112.83^a^ |  | 64.49^c^ |  |
| Hydrocarbons |  |  |  |  |  |  |  |  |
| 1-Tridecene | 0^b^ | 0 | 0.17^a^ | 0 - 0.68 | 0^b^ | 0 | 0.15^a^ | 0 - 0.72 |
| p-Menthene | 0^c^ | 0 | 0.28^b^ | 0 - 41.09 | 0^c^ | 0 | 0.65^a^ | 0 - 15.73 |
| 3,3-Dimethyl butanamide | 0^c^ | 0 | 0.08^b^ | 0 - 2.34 | 0^c^ | 0 | 0.21^a^ | 0.019 - 6.73 |
| β-Citronellol | 0.69^b^ | 0 - 336.44 | 0^c^ | 0 | 2.87^a^ | 0 - 8.42 | 0^c^ | 0 |
| 1,3-Di-tert-butylbenzene | 0^c^ | 0 - 210.04 | 0.49^b^ | 0 - 1.68 | 94.09^a^ | 0 - 182.08 | 0.73^b^ | 0 - 3.58 |
| α-Cubebene | 0.39^b^ | 0 - 25.26 | 0^c^ | 0 | 1.49^a^ | 0 - 3.42 | 0^c^ | 0 |
| Thiophene | 0^c^ | 0 - 12.47 | 1.65^a^ | 0 - 4.93 | 0^c^ | 0 - 2.81 | 0.03^b^ | 0 - 2.77 |

**Table 1S** continued

| **Chemical class** | **Treated coeliac children (T-CD)** | | | | **Healthy children (HC)** | | | |
| --- | --- | --- | --- | --- | --- | --- | --- | --- |
|  | **Faeces** | | **Urines** | | **Faeces** | | **Urines** | |
|  | Median | Range | Median | Range | Median | Range | Median | Range |
| 2,4-Dimethyl-1-Heptene | 0^b^ | 0 - 29.71 | 0^b^ | 0 - 2.14 | 1.73^a^ | 0 - 7.94 | 0^b^ | 0 - 4.69 |
| 3-(4-Methyl-3-pentenyl)furan | 0^a^ | 0 - 28.85 | 0^a^ | 0 | 0^a^ | 0 - 2.17 | 0^a^ | 0 |
| Ethyl-p-xylene | 0.46^b^ | 0 - 128.97 | 0.06^c^ | 0 - 1.85 | 6.12^a^ | 0.22 - 92.95 | 0.03^c^ | 0 - 0.50 |
| 5-Methyl-2-heptanone | 3.30^a^ | 0 - 26.15 | 0^c^ | 0 | 0.80^b^ | 0 - 5.62 | 0^c^ | 0 |
| Styrene | 2.23^a^ | 0 - 98.48 | 0.40^c^ | 0 - 1.62 | 1.48^b^ | 0 - 3.20 | 0.30^c^ | 0 - 1.19 |
| α-Pinene | 8.69^a^ | 0 - 172.64 | 0.53^b^ | 0 - 9.51 | 8.88^a^ | 0 - 285.06 | 0.50^b^ | 0.142 - 24.00 |
| β-Pinene | 0 | 0 - 179.67 | 0.23^bc^ | 0 - 1.02 | 0^c^ | 0 - 36.29 | 0.34^a^ | 0 - 2.36 |
| 3,5-Dimethyl octane | 0.93^b^ | 0 - 78.47 | 0^c^ | 0 - 0.21 | 1.67^a^ | 0 - 5.29 | 0^c^ | 0 - 0.29 |
| Total Hydrocarbons | 16.69^b^ |  | 4.25^c^ |  | 119.13^a^ |  | 3.14^c^ |  |
| Aldehydes |  |  |  |  |  |  |  |  |
| Benzaldehyde | 0^c^ | 0 | 0.88^b^ | 0 - 4.08 | 0^c^ | 0 | 2.43^a^ | 0 - 6.30 |
| 2-Ethyl benzaldehyde | 0^b^ | 0 | 51.65^a^ | 0 - 98.49 | 0^b^ | 0 | 52.86^a^ | 0 - 97.44 |
| Decanal | 0^b^ | 0 | 1.15^a^ | 0.18 - 2.37 | 0^b^ | 0 | 1.68^a^ | 0 - 2.86 |
| Hexanal | 0^b^ | 0 | 3.14^a^ | 0 - 10.01 | 0^b^ | 0 | 5.04^a^ | 0 - 33.11 |
| Furfural | 3.20^b^ | 0 - 20.89 | 0^c^ | 0 - 1.92 | 9.23^a^ | 0 - 58.38 | 1.18^b^ | 0 - 2.58 |
| Tetradecanal | 0^a^ | 0 - 129.16 | 0^a^ | 0 | 0^a^ | 0 - 8.33 | 0^a^ | 0 |
| Dodecanal | 0^b^ | 0 - 6.11 | 0^b^ | 0 | 0.74^a^ | 0 - 14.73 | 0^b^ | 0 |

**Table 1S** continued

| **Chemical class** | **Treated coeliac children (T-CD)** | | | | **Healthy children (HC)** | | | |
| --- | --- | --- | --- | --- | --- | --- | --- | --- |
|  | **Faeces** | | **Urines** | | **Faeces** | | **Urines** | |
|  | Median | Range | Median | Range | Median | Range | Median | Range |
| 3,5-Dimethyl benzaldehyde | 0^c^ | 0 - 31.21 | 0.44^b^ | 0.26 - 0.75 | 0.86^a^ | 0 - 14.19 | 0.41^b^ | 0 - 0.86 |
| Benzeneacetaldehyde | 0^b^ | 0 - 29.67 | 0^b^ | 0 | 2.23^a^ | 0 - 21.52 | 0^b^ | 0 |
| (E)-2-Nonenal | 0^b^ | 0 - 51.16 | 0^b^ | 0 | 1.33^a^ | 0 - 11.14 | 0^b^ | 0 |
| Nonanal | 12.58^a^ | 0 - 75.04 | 5.35^b^ | 0 - 37.83 | 17.01^a^ | 2.08 - 200.75 | 6.74^b^ | 0 - 43.43 |
| Octanal | 0^c^ | 0 - 9.39 | 1.32^b^ | 0 - 3.93 | 4.03^a^ | 0 - 22.51 | 2.55^b^ | 0.50 - 9.01 |
| 2-Methyl-butanal | 0^a^ | 0 - 35.02 | 0^a^ | 0 - 0.11 | 0^a^ | 0 - 2.51 | 0^a^ | 0 - 0.12 |
| Heptanal | 0.13^b^ | 0 - 9.55 | 0^c^ | 0 - 0.04 | 1.14^a^ | 0 - 3.94 | 0^c^ | 0 |
| 3-Methyl butanal | 1.09^a^ | 0 - 97.85 | 0^c^ | 0 - 4.58 | 0.89^b^ | 0 - 4.88 | 0^c^ | 0 - 0.29 |
| 2-Pentyl furan | 0.59^b^ | 0 - 17.23 | 0.36^b^ | 0 - 2.20 | 0^c^ | 0 - 2.37 | 0.75^a^ | 0 - 3.56 |
| Total aldehydes | 17.59^c^ |  | 64.31^a^ |  | 37.46^b^ |  | 73.37^a^ |  |
| Alcohols |  |  |  |  |  |  |  |  |
| Phenol | 0^a^ | 0 - 4.64 | 0^a^ | 0 | 0^a^ | 0 - 0 | 0^a^ | 0 |
| 4-Methyl phenol | 0^b^ | 0 | 0.15^a^ | 0 - 0.80 | 0^b^ | 0 | 0.20^a^ | 0 - 0.64 |
| 1-Tridecanol | 0^b^ | 0 | 0.33^a^ | 0 - 1.18 | 0^b^ | 0 | 0.25^a^ | 0 - 3.76 |
| 2-Methyl phenol | 0^b^ | 0 | 0^b^ | 0 - 0.37 | 0^b^ | 0 | 0.03^a^ | 0 - 0.99 |
| Phenylethanol | 0^b^ | 0 - 26.17 | 0^b^ | 0 | 2.87^a^ | 0 - 41.77 | 0^b^ | 0 |
| Tetradecanol | 37.09^b^ | 0 - 801.59 | 0^c^ | 0 | 51.30^a^ | 4.15 - 188.62 | 0^c^ | 0 |

**Table 1S** continued

| **Chemical class** | **Treated coeliac children (T-CD)** | | | | **Healthy children (HC)** | | | |
| --- | --- | --- | --- | --- | --- | --- | --- | --- |
|  | **Faeces** | | **Urines** | | **Faeces** | | **Urines** | |
|  | Median | Range | Median | Range | Median | Range | Median | Range |
| Tridecanol | 1.69^b^ | 0 - 154.31 | 0^c^ | 0 | 1.08^a^ | 0 - 5.31 | 0^c^ | 0 |
| 1-Octanol | 0^b^ | 0 - 95.25 | 0.85^a^ | 0.040 - 1.45 | 0^b^ | 0 - 2.11 | 0.65^a^ | 0 - 3.32 |
| 2-Nonanol | 0.22^a^ | 0 - 6.64 | 0^b^ | 0 - 0.49 | 0.88^a^ | 0 - 3.03 | 0.03^b^ | 0 - 1.42 |
| 1-Butanol | 0^c^ | 0 - 131.42 | 0.07^b^ | 0 - 1.26 | 15.32^a^ | 0 - 66.65 | 0.11^b^ | 0 - 0.35 |
| 1-Octen-3-ol | 82.81^a^ | 0 - 170.45 | 0.15^b^ | 0 - 1.64 | 0.62^b^ | 0 - 223.22 | 0.07^c^ | 0 - 0.88 |
| 2-Heptanol | 0.83^a^ | 0 - 17.85 | 0.35^b^ | 0 - 0.67 | 0.81^a^ | 0 - 12.80 | 0.24^b^ | 0 - 2.31 |
| Ethanol | 56.75^a^ | 0 - 350.32 | 0.36^c^ | 0 - 7.72 | 39.94^b^ | 0 - 141.74 | 0.29^c^ | 0 - 19.79 |
| 1-Propanol | 49.66^a^ | 0 - 503.47 | 0^c^ | 0 | 7.19^b^ | 4.83 - 177.42 | 0^c^ | 0 |
| Isoamyl alcohol | 1.09^ab^ | 0 - 49.18 | 0^c^ | 0 - 1.92 | 2.55^a^ | 0 - 71.55 | 0.26^b^ | 0 - 1.50 |
| Total Alcohols | 230.14^a^ |  | 2.25^c^ |  | 122.56^b^ |  | 2.14^c^ |  |
| Alkane |  |  |  |  |  |  |  |  |
| Hexadecane | 2.93^b^ | 0 - 71.93 | 0^c^ | 0 | 6.10^a^ | 0 - 151.25 | 0^c^ | 0 |
| 2-Dodecanone | 2.59^a^ | 0 - 537.44 | 0^b^ | 0 | 2.48^a^ | 0 - 278.38 | 0^b^ | 0 |
| Dodecane | 1.21^a^ | 0 - 12.81 | 0^c^ | 0 | 0.79^b^ | 0 - 2.29 | 0^c^ | 0 |
| Tetradecane | 0^b^ | 0 - 31.43 | 0.30^a^ | 0.05 - 1.57 | 0^b^ | 0 - 0.82 | 0.43^a^ | 0 - 1.47 |
| Total Alkane | 6.73^a^ |  | 0.3^b^ |  | 9.37^b^ |  | 0.43^c^ |  |
| Alkene |  |  |  |  |  |  |  |  |

**Table 1S** continued

| **Chemical class** | **Treated coeliac children (T-CD)** | | | | **Healthy children (HC)** | | | |
| --- | --- | --- | --- | --- | --- | --- | --- | --- |
|  | **Faeces** | | **Urines** | | **Faeces** | | **Urines** | |
|  | Median | Range | Median | Range | Median | Range | Median | Range |
| 1-Decene | 0^a^ | 0 - 32.51 | 0^a^ | 0 | 0^a^ | 0 - 31.99 | 0^a^ | 0 |
| Aromatic organic compounds |  |  |  |  |  |  |  |  |
| p-Cresol | 178.24^b^ | 0 - 977.15 | 0.10^c^ | 0 - 12.82 | 480.20^a^ | 233.74 - 993.94 | 0.57^c^ | 0 - 12.26 |
| Estragole | 0^a^ | 0 | 0^a^ | 0 - 0.77 | 0^a^ | 0 | 0^a^ | 0 - 0.32 |
| Toluene | 0^a^ | 0 | 0^a^ | 0 - 0.05 | 0^a^ | 0 | 0^a^ | 0 - 0.12 |
| 2-Ethenyl-6-methyl pyrazine | 0^b^ | 0 | 0.12^a^ | 0.04 - 12.04 | 0^b^ | 0 | 0.13^a^ | 0 - 0.76 |
| 4-Methyl thiazole | 0^c^ | 0 | 1.88^b^ | 0 - 2.48 | 0^c^ | 0 | 2.074^a^ | 0 - 2.84 |
| Indole | 0^a^ | 0 - 46.52 | 0^a^ | 0 | 0^a^ | 0 | 0^a^ | 0 |
| Total aromatic organic compounds | 178.24^b^ |  | 2.10^c^ |  | 480.20^a^ |  | 2.7842^c^ |  |
| Hetpane |  |  |  |  |  |  |  |  |
| 2,4-Dimethyl-Heptane | 23.01^a^ | 0 - 837.50 | 0^c^ | 0 - 1.37 | 26.37^a^ | 0 - 65.75 | 0.34^b^ | 0 - 2.37 |
| Short chain fatty acids (SCFA) |  |  |  |  |  |  |  |  |
| Octanoic acid | 0^a^ | 0 - 10.26 | 0^a^ | 0 | 0^a^ | 0 - 4.20 | 0^a^ | 0 |
| Isocaproic acid | 0^b^ | 0 - 3.93 | 0^b^ | 0 | 1.37^a^ | 0 - 14.59 | 0^b^ | 0 |
| Hexanoic acid | 0^b^ | 0 - 36.97 | 0^b^ | 0 - 0.34 | 0^b^ | 0 - 1.10 | 0.15^a^ | 0 - 4.59 |
| Pentanoic acid | 0^a^ | 0 - 202.69 | 0^a^ | 0 | 0^a^ | 0 - 119.91 | 0^a^ | 0 |

**Table 1S** continued

| **Chemical class** | **Treated coeliac children (T-CD)** | | | | **Healthy children (HC)** | | | |
| --- | --- | --- | --- | --- | --- | --- | --- | --- |
|  | **Faeces** | | **Urines** | | **Faeces** | | **Urines** | |
|  | Median | Range | Median | Range | Median | Range | Median | Range |
| Butyric acid | 0.83^b^ | 0 - 39.39 | 0^c^ | 0 | 1.23^a^ | 0 - 15.01 | 0^c^ | 0 |
| 3-Methyl butyric acid | 0^a^ | 0 - 53.24 | 0^a^ | 0 | 0^a^ | 0 - 28.65 | 0^a^ | 0 |
| Isobutyric acid | 1.36^a^ | 0 - 21.80 | 0^b^ | 0 | 0^b^ | 0 - 6.87 | 0^b^ | 0 |
| Propanoic acid | 0.88^c^ | 0 - 161.26 | 2.20^b^ | 0.02 - 5.20 | 21.54^a^ | 0 - 289.81 | 2.30^b^ | 1.40 - 3.92 |
| Acetic acid | 18.48^a^ | 0 - 778.62 | 0.39^c^ | 0 - 13.20 | 2.86^b^ | 0 - 547.18 | 0.36^c^ | 0 - 2.02 |
| 2-Methyl butyric acid | 0^c^ | 0 | 0.40^b^ | 0.06 - 12.40 | 0^c^ | 0 | 1.01^a^ | 0.04 - 14.34 |
| Hexyl valerate | 0.09^b^ | 0 - 140.38 | 0^c^ | 0 | 0.85^a^ | 0 - 14.38 | 0^c^ | 0 |
| Total short chain fatty acids (SCFA) | 21.64^a^ |  | 3^b^ |  | 27.85^a^ |  | 3.82^b^ |  |

Data are the means of three independent experiments (n = 3) for each children.

^a-c^Means within a row with different superscript letters are significantly different (*P*<0.05).
